# Social cognition and social motivation in schizophrenia and bipolar disorder: are impairments linked to the disorder or to being socially isolated?

**DOI:** 10.1017/S0033291724000102

**Published:** 2024-07

**Authors:** Michael F. Green, Jonathan K. Wynn, Naomi I. Eisenberger, William P. Horan, Junghee Lee, Amanda McCleery, David J. Miklowitz, Eric A. Reavis, L. Felice Reddy

**Affiliations:** 1 Department of Psychiatry and Biobehavioral Sciences, Jane and Terry Semel Institute for Neuroscience and Human Behavior, UCLA, Los Angeles, CA, USA; 2 VA Greater Los Angeles Healthcare System, Los Angeles, CA, USA; 3 VA Rehabilitation R&D Center on Enhancing Community Integration for Homeless Veterans, Los Angeles, CA, USA; 4 Department of Psychology, UCLA, Los Angeles, CA, USA; 5 Karuna Therapeutics, Boston, MA, USA; 6 Department of Psychiatry and Behavioral Neurobiology, University of Alabama at Birmingham, Birmingham, AL, USA; 7 Department of Psychological and Brain Sciences, University of Iowa, Iowa City, IA, USA; 8 Department of Psychiatry, University of North Carolina, Chapel Hill, NC, USA

**Keywords:** bipolar disorder, schizophrenia, social cognition, social isolation, social motivation

## Abstract

**Background:**

People with schizophrenia on average are more socially isolated, lonelier, have more social cognitive impairment, and are less socially motivated than healthy individuals. People with bipolar disorder also have social isolation, though typically less than that seen in schizophrenia. We aimed to disentangle whether the social cognitive and social motivation impairments observed in schizophrenia are a specific feature of the clinical condition *v.* social isolation generally.

**Methods:**

We compared four groups (clinically stable patients with schizophrenia or bipolar disorder, individuals drawn from the community with self-described social isolation, and a socially connected community control group) on loneliness, social cognition, and approach and avoidance social motivation.

**Results:**

Individuals with schizophrenia (*n* = 72) showed intermediate levels of social isolation, loneliness, and social approach motivation between the isolated (*n* = 96) and connected control (*n* = 55) groups. However, they showed significant deficits in social cognition compared to both community groups. Individuals with bipolar disorder (*n* = 48) were intermediate between isolated and control groups for loneliness and social approach. They did not show deficits on social cognition tasks. Both clinical groups had higher social avoidance than both community groups

**Conclusions:**

The results suggest that social cognitive deficits in schizophrenia, and high social avoidance motivation in both schizophrenia and bipolar disorder, are distinct features of the clinical conditions and not byproducts of social isolation. In contrast, differences between clinical and control groups on levels of loneliness and social approach motivation were congruent with the groups' degree of social isolation.

## Introduction

Individuals with schizophrenia experience long-standing disability in multiple social domains; in fact, social disability is a defining aspect of the condition (APA, 2013; WHO, 2008). One key component of social disability in schizophrenia is social isolation, defined as the objective lack of peer and family relationships and minimal participation in community activities (Green et al., 2018; Necka, Rowland, & Evans, 2021). Objective social isolation is distinct from loneliness, which is the subjective discomfort of feeling insufficiently connected to others (Hawkley & Cacioppo, 2010). Social isolation is, of course, not solely a feature of schizophrenia; it occurs with surprising frequency in the general community where it represents a substantial public health concern (Holt-Lunstad, Robles, & Sbarra, 2017; Holt-Lunstad, Smith, Baker, Harris, & Stephenson, 2015; Wang et al., 2023). It also occurs at moderate levels in bipolar disorder and other mood disorders (WHO, 2008).

Overall, the personal factors underlying social disability and isolation in schizophrenia can be divided into two general categories: social cognition and social motivation. Social cognition refers to one's capacity to process social information and includes the ability to understand emotions in faces, infer what other people are thinking and feeling, regulate one's own emotions, and monitor moment-to-moment changes in another's mood (Green et al., 2008; Green, Horan, & Lee, 2019; Kunda, 1999). Social cognition can be divided into separate domains, including social cue perception (including face affect identification), mentalizing (also called theory of mind), and integrative processes (e.g. empathy). Individuals with schizophrenia usually show impairment in all of these social cognitive domains (Green, Horan, & Lee, 2015; Savla, Vella, Armstrong, Penn, & Twamley, 2013).

In contrast to social cognition, social motivation refers to one's desire to engage in social activities and the perceived reward, or threat, of social interactions (Catalano & Green, 2023; Fulford, Campellone, & Gard, 2018a; Fulford, Treadway, & Woolley, 2018b). Social motivation can be divided into two processes that involve distinct neural systems: social approach motivation (desire to be with other people) and social avoidance motivation (desire to be away from other people) (Barch & Dowd, 2010; Baumeister & Leary, 1995; Chang et al., 2013). Schizophrenia is associated with deficits in both types of social motivation. Historically, the focus of research on social interactions has been on social anhedonia, which is the same as low social approach motivation (Catalano & Green, 2023; Meehl, 2001).

Hence, there is abundant evidence from multiple sources that people with schizophrenia have more social isolation, more social cognitive impairment, and abnormalities in social motivation compared with healthy controls. However, a fundamental knowledge gap remains: we do not know whether the social cognitive and social motivation deficits in schizophrenia are associated with the clinical condition itself, or whether they are secondary to the effects of social isolation. When studies compare people with schizophrenia to healthy controls, they typically try to match groups on age, gender, and parental education, but rarely account for differences in levels of social isolation. Thus, previously reported differences observed between patient and control groups in social cognition or social motivation may reflect the effects of the clinical disorder or they could be effects more generally related to social isolation, nonspecific to schizophrenia.

Bipolar disorder is an informative comparison sample for schizophrenia, as well as an important clinical focus of study itself. It is also a chronic psychiatric condition that relapses and remits, although with lower reported levels of impairment in social cognition and social motivation compared to those with schizophrenia (Bora, Yucel, & Pantelis, 2009; Lee et al., 2013). Similarly, social functioning in bipolar disorder, at the group level, is typically below that of healthy controls, and above that of schizophrenia (Gitlin & Miklowitz, 2017; Harrow, Grossman, Herbener, & Davies, 2000). As in schizophrenia, we do not know whether deficits (if observed) in social cognition or social motivation in bipolar disorder reflect the clinical condition itself, or a history of social isolation.

The focus of the current study was to better understand whether social cognition or motivation are attributable to disorder-specific features in schizophrenia and bipolar disorder, or whether they are more generally related to social isolation. Specifically, the goal was to determine whether either clinical group would show deficits on social measures relative to a community comparison group selected for high levels of social isolation or a typical healthy control group. Significant impairments in a clinical sample relative to the socially isolated community group would suggest the impairment is attributable to the clinical condition. By contrast, deficits relative to the healthy control group, but not the socially isolated community group, could be attributable to social isolation, independent of the clinical disorders.

Based on existing data, we expected the schizophrenia group to have more impairment in social cognition and social motivation compared with the non-isolated control group, with intermediate impairment in the bipolar group. We are not aware of any previous comparison of schizophrenia and bipolar disorder to a socially isolated community sample; thus, we did not have a basis to predict how the clinical groups would differ from the isolated group.

## Methods

### Participants

This study included 72 outpatients with schizophrenia, 48 with bipolar disorder, and 151 individuals from the community. The clinical groups were recruited from outpatient clinics at the Veterans Affairs Greater Los Angeles Healthcare System (GLA) and the University of California, Los Angeles (UCLA), and outpatient board and care facilities in the Los Angeles area. Psychiatric diagnoses were established with the Structured Clinical Interview for DSM-5 (SCID-5) from Modules A-E and the PTSD section (First, Williams, Karg, & Spitzer, 2015b). When they were available, medical records were examined to corroborate information from interviews. All clinical participants were clinically stable, with no hospitalizations within three months and no changes in psychoactive medication type/dosage within four weeks. Both patient groups were receiving psychoactive medications at the time of assessment.

To recruit a community sample high in social isolation, we placed advertisements online (Craigslist) that asked: ‘Do you have few friends, little contact with family members, and typically do activities alone?’ We recruited 96 subjects from the community through these ads. We also ran ads on the same website that were similar to those used in our previous studies in which we asked for control participants but did not mention anything about social connections. Fifty-five participants responded to these ads. The community isolated group was comprised of participants who responded to the first ad, self-identifying as having few friends or little contact with family. The community control group was comprised of participants who responded to the second ad that did not mention social contacts.

All community participants provided psychiatric history through the SCID-5 and select sections of the SCID for Personality Disorders (SCID-PD) assessing avoidant, paranoid, schizoid, schizotypal, and borderline characteristics, (First, Williams, Benjamin, & Spitzer, 2015a) and were excluded if they met criteria for a lifetime history of a psychotic disorder or bipolar disorder. Personality disorder diagnoses were not exclusionary in community groups. All study procedures were approved by the Institutional Review Boards of GLA and UCLA. All participants had the capacity to give informed consent and provided informed consent prior to participation after all procedures were fully explained.

Inclusion criteria for all participants were: (a) age 20–60, (b) understanding of English to a sufficient level to comprehend procedures, (c) no clinically significant neurological disease (e.g. epilepsy), (d) no history of a serious head injury (loss of consciousness > 1 h), (e) no sedatives or benzodiazepines within 12 h of testing, (f) no evidence of IQ < 70 or developmental disability based on the Wide-Range Achievement Test 3rd ed. reading subtest (Wilkinson, 1993), (g) no substance use disorder at moderate level or greater in the past three months, and (h) no current mood episode meeting clinical criteria for depression, hypomania, or mania.

Social isolation was rated with a standardized composite of three complementary scales: (1) Lubben Social Network Scale (12 item version) (Lubben, 1988), (2) Social Disconnectedness Scale (last 4 items) (Cornwell & Walte, 2009), and (3) the Role Functioning Scale (social and family scores) (McPheeters, 1984). A preliminary reliability analysis from an unpublished data set showed that combining these three scores yielded a homogeneous scale (Cronbach's alpha = 0.887). Communalities range between 0.931 and 0.665, showing that items cover different facets of the construct and were not redundant. The composite score was based on the data from the control (non-isolated) community sample only. We created the composite score by separately norming each of the three scales (respective to the control community sample), taking the average of the normed scores, and then taking the inverse of the score. Thus, larger values on the composite score indicate greater isolation.

### Assessments

All assessments on individuals from the community and the schizophrenia samples were completed in person, prior to the COVID-19 pandemic. Most assessments of the bipolar group were conducted during the same period, but a small number of participants in that group (*n* = 7) were recruited later and assessed in person after the end of pandemic-related restrictions.

### Diagnostic interviews and other self-report scales

Beyond the diagnostic interviews, clinical symptom ratings were conducted for all clinical participants with the Expanded Brief Psychiatric Rating Scale (BPRS) (Ventura et al., 1993), the Hamilton Depression Scale (Hamilton, 1960), the Young Mania Rating Scale (Young, Biggs, Ziegler, & Meyer, 1978) and the Clinical Assessment Interview for Negative Symptoms (CAINS) (Blanchard et al., 2010). Training and quality assurance on all interviews were conducted through established procedures by the Treatment Unit of the VA VISN 22 Mental Illness Research Education and Clinical Center (MIRECC).

We included the UCLA Loneliness Scale (Russell, 1996) to assess loneliness (higher score indicates greater loneliness) and the Short Autism Spectrum Scale (AQ-10) (Allison, Auyeung, & Baron-Cohen, 2012). A comparison of adults with autism spectrum disorder and controls using the AQ-10 indicated that a cut-off score of 6 or greater identified people in the autism spectrum (Allison et al., 2012).

### Social cognition

#### Mentalizing: the awareness of social inference test (TASIT) – Part 3

On this measure, participants watched a series of videotaped vignettes that depict people interacting and answered four types of questions about what a person in the conversation: (a) believes or knows, (b) means, (c) intends, and (d) feels (McDonald, Flanagan, & Rollins, 2002). Part 3 of the TASIT assesses the ability to use contextual knowledge (visual and verbal) in addition to voice and face cues to derive meaning from the conversation. It includes 16 vignettes in which there is an untrue comment presented as either sarcasm or as a lie. This task has good psychometric properties (McDonald et al., 2006). A higher score indicates better mentalizing performance (range 0–64).

#### Empathic accuracy task

We have used variations of this task in previous studies of severe mental illness and comparison samples (Harvey, Zaki, Lee, Ochsner, & Green, 2013; Kern et al., 2013; Lee, Zaki, Harvey, Ochsner, & Green, 2011). Participants watched clips lasting 2.0–2.5 min that show an individual (a ‘target’) while he/she discusses a positive or negative autobiographical event. Participants used response keys to continuously rate how positive or negative they believe the target was feeling throughout each clip. There were a total of nine clips. The dependent measure is the mean correlation across clips between the participant's ratings of the targets' emotions and targets' ratings of their own emotions, with a higher correlation indicating greater empathic accuracy (range: 0–1).

#### Facial affect identification test

In this test, participants identified facial expressions of seven different emotions in still color photographs from a standardized stimulus set by Ekman (Ekman, 1976). This test demonstrates good psychometric properties (Horan et al., 2011; Kern et al., 2013), and shows relationships to functioning (Olbert et al., 2013). A higher score indicates better affect identification (range: 0–56).

### Social motivation

Social approach motivation was assessed with a scale that is commonly used in schizophrenia research, the Social Anhedonia Scale – Brief (SAS) (Reise, Horan, & Blanchard, 2011). The SAS is a 24-item (dichotomously scored) self-report measure for assessing decreased social pleasure, including lack of interest in social connections, aversion from social interactions, and preference for solitude and solo activities. A lower score on the SAS means greater social approach.

For social avoidance we used a scale with established psychometric properties: the Sensitivity to Rejection Scale (Mehrabian, 1972; Mehrabian, 1976). The Sensitivity to Rejection scale contains 24 items which represent the following factors: avoidance of behaviors or situations involving arguments or critical interaction (‘I criticize people openly and expect them to do the same’ – reverse scored); fear of expressing personal opinions when these might be rejected (‘When a group is discussing an important matter, I like my feelings to be known’ – reverse scored); timidity in situations where there is the slightest possible hint of rejection (‘I often visit people without being invited’ – reverse scored); being easily hurt by negative feedback from others and fearing such feedback (‘I would be very hurt if a close friend should contradict me in public’); and reliance on familiar others as a means of avoiding rejection (‘I sometimes prefer being with strangers than with familiar people’ – reverse scored) (Mehrabian, 1976). A higher score on the Sensitivity to Rejection Scale indicates greater social avoidance (range −96 – +96).

### Data analysis

All analyses were conducted using SPSS v27. Figures were generated with R (R Core Team, 2021) using the ggplot2 package version 3.3.5 (Wickham, 2016). We did not consider social isolation as one of the main outcome variables. Rather, we compared the social isolation data across groups to confirm expected differences in isolation level. For the six main outcome variables (loneliness, three social cognitive measures, two social motivation measures), we used one-way analysis of variance (ANOVA) tests and set the alpha threshold to 0.0083 (*p* = 0.05/6). Additionally, as there were significant group differences in age, we included age as a covariate in the six main ANOVAs. Effect sizes for ANOVAs are presented as partial eta-squared (*η*_p_^2^) values. Significant effects were followed up with Least Significant Difference pairwise comparisons. Data for the social cognition and social motivation measures are presented as box plots with individual data points overlaid. A small percentage of participants did not complete all measures, thus there are minor variations in the degrees of freedom across measures.

For clinical data, we compared the two clinical groups on symptom rating scales using independent samples *t* tests. Unless otherwise noted, data presented in the tables are mean (standard deviation) or total n's (%). Tallies are provided for the number of people in each community sample who were diagnosed with a personality disorder and the number who scored above threshold on an autism scale. The specific types of personality disorder for each group are listed as follows:

Isolated group (total with PDs = 24): 11 avoidant, 1 borderline, 4 paranoid, 4 schizoid, 2 avoidant and schizoid, 1 paranoid and schizotypal, 1 schizoid and paranoid. Control group (total with PDs = 3): 1 schizotypal, 1 schizoid, 1 avoidant.

## Results

The demographic and clinical data for the four groups are shown in Table 1. For any group differences the pairwise contrasts are shown in the right-hand column.

**Table 1.** Demographics and clinical information for each of the four groups

	Schizophrenia (SZ; *n* = 72)	Bipolar (BD; *n* = 48)	Isolated community (IC; *n* = 96)	Controls (CTL; *n* = 55)	Statistical test (*F, t*, or χ^2^)
Age	47.4 (11.3)	45.5 (10.8)	44.3 (11.0)	49.7 (8.1)	*F*_3267_ = 3.47, *p* = 0.017 CTL > BD, IC
Personal education	12.9 (1.7)	14.5 (2.0)	14.8 (2.1)	15.1 (2.1)	*F*_3267_ = 16.30, *p* < 0.001 SZ < BD, CTL, IC
Parental education	13.8 (3.4)	15.1 (2.9)	14.2 (2.8)	14.6 (3.1)	*F*_3251_ = 1.64, *p* < 0.19 SZ < BD
Sex (Male:Female)	47:25	29:19	56:40	41:14	χ^2^_(3)_ = 4.30, *p* = 0.23
BPRS positive	2.0 (0.9)	1.3 (0.3)			*t*_113_ = 5.37, *p* < 0.001
CAINS MAP	1.7 (0.9)	1.3 (0.8)			*t*_115_ = 2.55, *p* < 0.02
CAINS EXP	1.0 (0.9)	0.5 (0.7)			*t*_118_ = 3.12, *p* < 0.002
YMRS	5.9 (5.5)	3.7 (4.8)			*t*_118_ = 2.31, *p* < 0.03
HAMD	7.3 (5.4)	8.1 (6.2)			*t*_117_ = 0.76, *p* < 0.45
Personality disorder (people with 1 or more)			*N* = 24	*N* = 3	
AQ10 (score of 6 or higher)			*N* = 6	*N* = 1	

BPRS positive, Expanded Brief Psychiatric Rating Scale, positive symptom subscore.

CAINS MAP, Clinical Assessment Interview for Negative Symptoms, Motivation and Pleasure.

CAINS EXP, Clinical Assessment Interview for Negative Symptoms: Expressive.

YMRS, Young Mania Rating Scale.

HAMD, Hamilton Depression Scale.

AQ, Short Autism Spectrum Scale.

Data presented are mean (standard deviation) unless otherwise noted.

**Figure 1.**
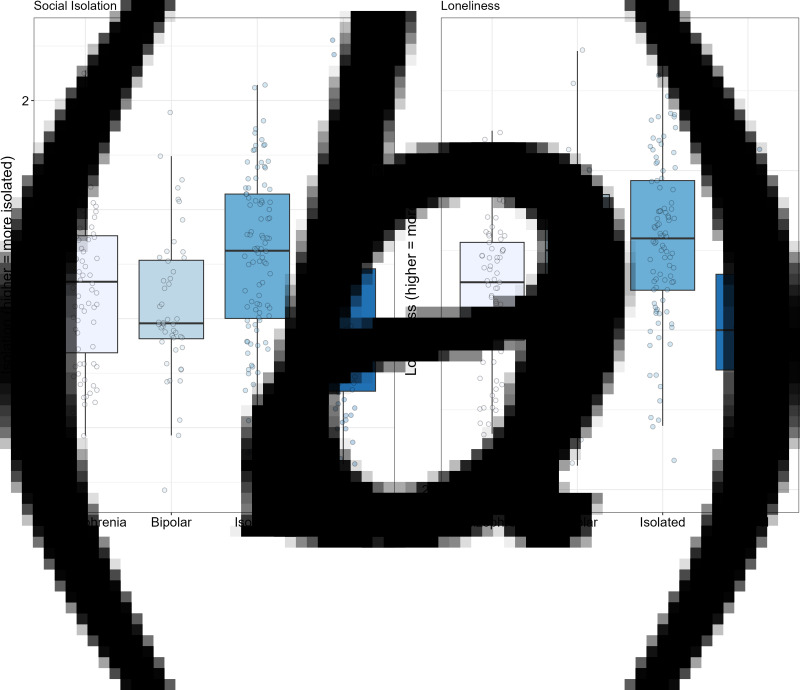
Box plots for objective social isolation (Panel A) and for loneliness (i.e. subjective social isolation) (Panel B). Solid black horizontal line indicates the median. Each dot represents the score for an individual within that group.

**Figure 2.**
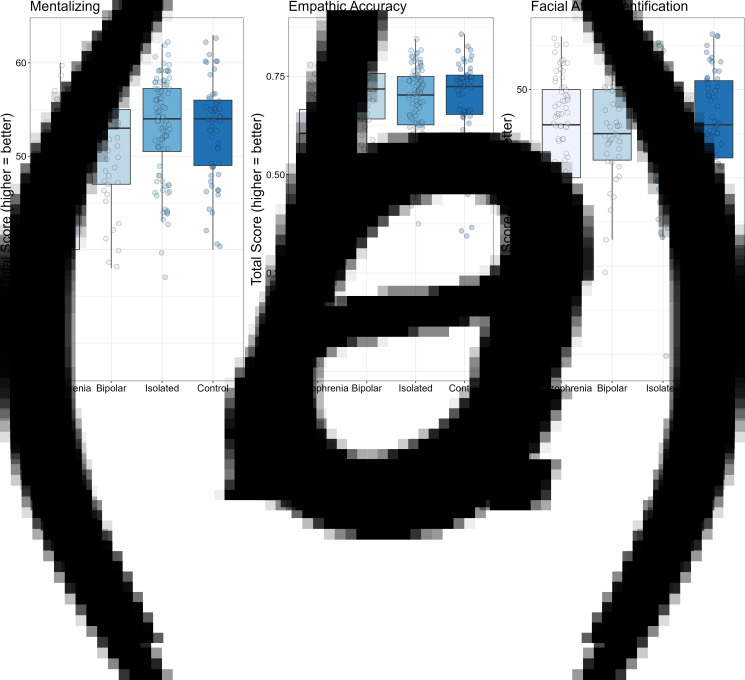
Box plots for mentalizing (TASIT; Panel A), empathic accuracy (Panel B), and facial affect identification (Panel C). For all tasks, higher scores indicate better performance. Solid black horizontal line indicates the median. Each dot represents the score for an individual within that group.

**Figure 3.**
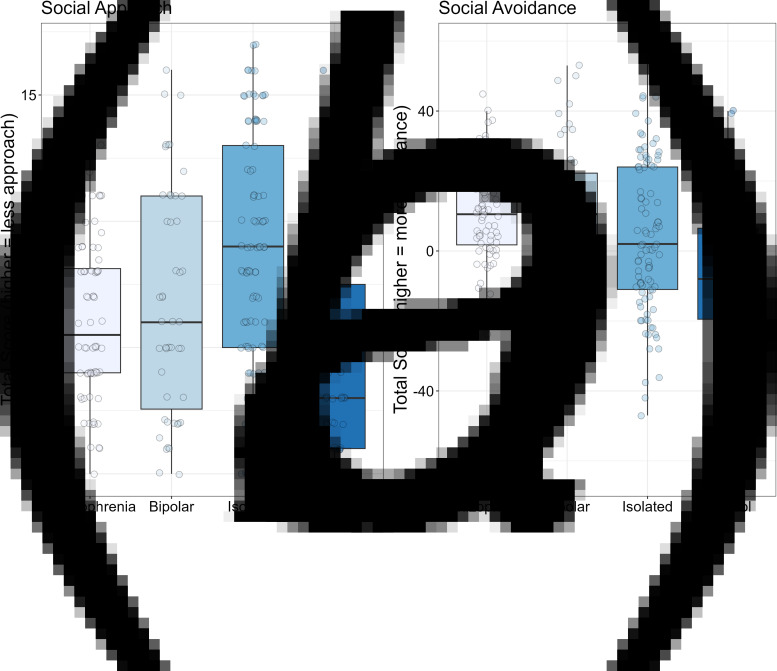
Box plots for social approach motivation (Social Anhedonia Scale; Panel A) and for social avoidance motivation (Sensitivity to Rejection Scale; Panel B). Solid black horizontal line indicates the median. Each dot represents the score for an individual within that group.

The data for the key dependent measures are shown in Table 2 and in Figs 1–3.

**Table 2.** Descriptive statistics and statistical test summary for the key variables

	Schizophrenia	Bipolar	Isolated	Control	*F* test, pairwise comparisons
Composite	0.31 (0.74)	0.12 (0.66)	0.55 (0.77)	0.00 (0.90)	*F*_3266_ = 7.87, *p* < 0.01, *η*^2^_p_ = 0.08 SZ > CTL; IC > SZ, BD, CTL
Loneliness	44.44 (9.61)	49.11 (10.84)	51.03 (10.47)	40.22 (10.40)	*F*_3263_ = 14.12, *p* < 0.01, *η*^2^_p_ = 0.14 SZ < BD, IC; CTL < SZ, IC, BD
TASIT total	45.52 (7.25)	51.60 (6.14)	53.03 (5.47)	52.57 (5.52)	*F*_3255_ = 22.95, *p* < 0.01, *η*^2^_p_ = 0.21 SZ < BD, IC, CTL
Empathic accuracy	0.57 (0.16)	0.70 (0.09)	0.68 (0.10)	0.69 (0.11)	*F*_3239_ = 14.81, *p* < 0.01, *η*^2^_p_ = 0.16 SZ < BD, IC, CTL
Facial affect	44.53 (7.65)	44.98 (5.34)	46.36 (5.72)	45.76 (7.09)	*F*_3252_ = 1.09, *p* < 0.36, *η*^2^_p_ = 0.01
Social approach	6.14 (3.40)	6.80 (4.53)	8.64 (4.73)	4.48 (4.63)	*F*_3264_ = 10.70, *p* < 0.01, *η*^2^_p_ = 0.11 SZ, BD > CTL; SZ < IC; IC > BD, CTL
Social avoidance	10.46 (13.76)	12.31 (19.91)	4.15 (22.41)	−7.11 (20.96)	*F*_3265_ = 12.13, *p* < 0.01, *η*^2^_p_ = 0.12 SZ, BD > CTL, IC; IC > CTL

TASIT, The awareness of social inference test.

SZ, schizophrenia group.

BD, bipolar group.

IC, isolated group.

CTL, control group.

All values are mean (standard deviation). Effect size is partial eta-squared (*η*^2^_p_). Note that age was included as a covariate in each ANOVA.

The composite score for social isolation is shown in Panel A of Fig. 1. The ANOVA was significant, *F*_3266_ = 7.87, *p* < 0.001, *η*^2^_p_ = 0.08. Looking at paired contrasts, the schizophrenia group was significantly more isolated than the control group (*p* < 0.02), and significantly less isolated than the isolated community group (*p* < 0.05) but did not differ from the bipolar group (*p* = 0.17). As expected, the isolated community group was more isolated than the control group and the bipolar group (*p*'s < 0.002). There was no significant difference in isolation between the bipolar and control groups (*p* = 0.42).

Loneliness (Panel B, Fig. 1) also showed a significant difference among the groups, *F*_3263_ = 14.12, *p* < 0.001, *η*^2^_p_ = 0.14. Paired contrasts showed that the participants with schizophrenia were significantly less lonely than participants in the isolated community and bipolar groups (*p*'s < 0.02), but significantly more lonely than the controls (*p* < 0.03). The isolated community group was significantly more lonely than the controls (*p* < 0.001), but did not differ from the bipolar group (*p* = 0.30). Finally, the bipolar group had significantly more loneliness than the control community group (*p* < 0.001).

The results for the three social cognitive measures are shown in Fig. 2. For the mentalizing task (i.e. TASIT) shown in Panel A, there was a significant difference among the groups, *F*_3255_ = 22.95, *p* < 0.001, *η*^2^_p_ = 0.21. The age covariate was significant, *F*_1255_ = 3.95, *p* < 0.05, *η*^2^_p_ = 0.02. The schizophrenia group showed significant deficits compared to the isolated, control, and bipolar groups (all *p*'s < 0.001); there were no other significant group differences (all *p*'s > 0.19). The pattern was similar for Empathic Accuracy Task (Panel B), *F*_3239_ = 14.81, *p* < 0.001, *η*^2^_p_ = 0.16. Again, the schizophrenia group showed lower scores compared to all three other groups (all *p*'s < 0.001), with no other significant group differences (all *p*'s > 0.45). For Facial Affect Identification (Panel C), no significant group differences were detected, *F*_3252_ = 1.09, *p* < 0.36, *η*^2^_p_ = 0.01.

The social approach and avoidance results are shown in Fig. 3. For social approach motivation (Panel A in which lower scores are better), there was a significant group effect, *F*_3264_ = 10.70, *p* < 0.001, *η*^2^_p_ = 0.11. The schizophrenia group showed significantly more approach motivation compared to the isolated community group (*p* < 0.001), significantly less approach motivation than controls (*p* < 0.04), and no difference compared to the bipolar group (*p* < 0.43). The isolated community group also showed significantly less approach motivation compared to the control and bipolar groups (*p*'s < 0.02). The bipolar group had significantly less approach motivation than controls (*p* < 0.01).

For social avoidance motivation, there was a significant group effect, *F*_3265_ = 12.13, *p* < 0.001, *η*^2^_p_ = 0.12. The age covariate was significant, *F*_1265_ = 5.84, *p* < 0.02, *η*^2^_p_ = 0.02. The schizophrenia group showed more avoidance than the control (*p* < 0.001) and isolated community groups (*p* < 0.05), but did not differ from the bipolar group (*p* < 0.62). The isolated group showed significantly more avoidance than controls (*p* < 0.001) but significantly less than the bipolar group (*p* < 0.02). Additionally, the bipolar group showed significantly more avoidance motivation than controls (*p* < 0.01).

## Discussion

We compared four groups (clinically stable individuals with schizophrenia or bipolar disorder, community members who self-identified as socially isolated, and a community control group) on loneliness, performance-based social cognition, and social motivation (approach and avoidance). The focus of these analyses was to better understand group differences and to determine whether deficits on these factors in schizophrenia and bipolar disorder were attributable to the clinical conditions themselves, or more broadly related to social isolation separate from psychopathology.

We found clear patterns for the variables, and they were somewhat different for the schizophrenia *v.* bipolar disorder groups. Individuals with schizophrenia were significantly more isolated than the control group, but less isolated than the isolated community group. Similarly, people in the schizophrenia group were lonelier than the control group, but less lonely than the isolated group. Social approach motivation showed a similar pattern to loneliness: patients had less social motivation than controls but more than the isolated group. Thus, deficits in loneliness and social approach motivation appeared to be tied to social isolation rather than schizophrenia *per se*.

In contrast, individuals in the schizophrenia group showed clear impairments on two of the three social cognitive measures compared to both community groups. Hence, these deficits appear to be linked to the disorder itself and not broadly to social isolation. Further supporting this interpretation, the isolated and control community groups did not differ from each other on any social cognitive measure. Similarly, the schizophrenia group differed from both community groups in social avoidance, indicating that group differences in avoidance motivation are linked to the clinical condition.

Individuals in the bipolar group reported significantly more loneliness than the control group and the schizophrenia group, whereas they did not differ from the isolated group. Emotional reactions to social isolation may be more salient and cognitively accessible in patients with bipolar disorder (Lee et al., 2013; Ng & Johnson, 2013). This group may be more aware of prior experiences with social isolation and the associated emotional states – such as loneliness – than patients with schizophrenia. Our results are consistent with findings that people with bipolar disorder report feeling more lonely than the general population, and loneliness was both a precursor and potentially a risk factor for recurrences of mood episodes, suicidal behavior, low self-rated health and poorer quality of life in mood disorders (Giacco, 2023).

Unlike schizophrenia, the bipolar group did not show deficits in social cognition compared with either community group. This finding is consistent with previous observations that social cognition is relatively intact in bipolar disorder compared with schizophrenia (Bora & Pantelis, 2016; Gillissie et al., 2022; Lee et al., 2013).

The bipolar group showed significantly more approach motivation than the isolated group and less than the control group, a pattern congruent with differences in the groups' levels of social isolation. In contrast, the bipolar group had more social avoidance than either community group. This pattern suggests that high social avoidance in bipolar disorder is attributable to the clinical condition. At least one study has shown that patients with bipolar disorder are more sensitive to social rejection than healthy controls (Ng & Johnson, 2013) which may be correlated with more social avoidance.

Because social cognition for schizophrenia and social avoidance for both schizophrenia and bipolar disorder appear to be directly tied to the clinical conditions, they are rational targets for intervention. Social cognition interventions are applied to schizophrenia using training approaches, as well as augmentation with psychopharmacology. Social avoidance is a treatment target for psychosocial interventions, such as social skills training, Motivational Interviewing, or Cognitive Behavioral Therapy. If treatments bring about reductions in rejection sensitivity, people with these conditions may become more open to establishing and maintaining social connections. These results reinforce the idea that a focused consideration of an individual's social and interpersonal context should be an important part of their treatment.

This study had several limitations. It was cross-sectional and therefore could not address important longitudinal questions (e.g. how the relationships among variables might change over time). Our measure of social avoidance motivation (Sensitivity to Rejection Scale) measured discomfort, fear, or concern about social interactions. However, we lacked a measure of real-world social rejection. Also, the social cognition measures were performance-based tasks whereas the social motivation measures were self-report scales. A more direct comparison of social domains could be conducted with validated performance-based measures of social motivation, though few exist. Lastly, the isolated group was more isolated than either patient group; it would be valuable for future studies to match clinical and nonclinical groups on level of isolation.

Despite these limitations, the current analyses shed light on an interpretive problem that has confounded research on social processes and disability in schizophrenia and bipolar disorder. By using a socially isolated community comparison group, we aimed to disentangle which impairments are core features of the disorders even when controlling for potential effects attributable to the social isolation that frequently occurs in these illnesses. We found group differences in loneliness and social approach motivation that were congruent with the groups' levels of social isolation. Therefore, we conclude that lower scores on these factors in schizophrenia and bipolar disorder may not be driven by illness-specific factors. However, our results suggest that impaired social cognition in schizophrenia, and elevated social avoidance in both schizophrenia and bipolar disorder are, in fact, features of those clinical conditions, because they do not occur to the same extent in a community sample with even higher levels of social isolation.
